# Effects of Whole and Partial Heart Irradiation on Collagen, Mast Cells, and Toll-like Receptor 4 in the Mouse Heart

**DOI:** 10.3390/cancers15020406

**Published:** 2023-01-07

**Authors:** Vijayalakshmi Sridharan, Kimberly J. Krager, Snehalata A. Pawar, Shivani Bansal, Yaoxiang Li, Amrita K. Cheema, Marjan Boerma

**Affiliations:** 1 Division of Radiation Health, University of Arkansas for Medical Sciences, Little Rock, AR 72205, USA; 2 Department of Radiation Oncology, Upstate Cancer Center, Upstate Medical University, Syracuse, NY 13210, USA; 3 Department of Radiation Oncology, Georgetown University, Washington, DC 20057, USA

**Keywords:** radiation-induced heart disease, partial heart irradiation, mouse model, Toll-like receptor 4

## Abstract

**Simple Summary:**

In radiation therapy of tumors in the chest, such as in lung or esophageal cancer, part of the heart may be situated in the radiation field. This can lead to the development of radiation-induced heart disease. The mechanisms by which radiation causes a long-term injury to the heart are not fully understood, but investigations in pre-clinical research models can contribute to mechanistic insights. To model partial heart irradiation as it may occur in patients, in this study, adult male and female mice were exposed to irradiation to only 40% of the heart. Mice exposed to a whole heart irradiation were used for comparison. While plasma samples at 5 days and 2 weeks after the irradiation showed different metabolite profiles, we found no differences in the tissue structural changes between the irradiated and unirradiated portions of the heart at 6 months. Additional work in larger animal cohorts is required to determine whether there are differences between the two sexes.

**Abstract:**

In radiation therapy of tumors in the chest, such as in lung or esophageal cancer, part of the heart may be situated in the radiation field. This can lead to the development of radiation-induced heart disease. The mechanisms by which radiation causes long-term injury to the heart are not fully understood, but investigations in pre-clinical research models can contribute to mechanistic insights. Recent developments in X-ray technology have enabled partial heart irradiation in mouse models. In this study, adult male and female C57BL/6J mice were exposed to whole heart (a single dose of 8 or 16 Gy) and partial heart irradiation (16 Gy to 40% of the heart). Plasma samples were collected at 5 days and 2 weeks after the irradiation for metabolomics analysis, and the cardiac collagen deposition, mast cell numbers, and left ventricular expression of Toll-like receptor 4 (TLR4) were examined in the irradiated and unirradiated parts of the heart at 6 months after the irradiation. Small differences were found in the plasma metabolite profiles between the groups. However, the collagen deposition did not differ between the irradiated and unirradiated parts of the heart, and radiation did not upregulate the mast cell numbers in either part of the heart. Lastly, an increase in the expression of TLR4 was seen only after a single dose of 8 Gy to the whole heart. These results suggest that adverse tissue remodeling was not different between the irradiated and unirradiated portions of the mouse heart. While there were no clear differences between male and female animals, additional work in larger cohorts may be required to confirm this result, and to test the inhibition of TLR4 as an intervention strategy in radiation-induced heart disease.

## 1. Introduction

Radiation therapy (RT) is used to treat more than 70% of all cancer types [1]. Improvements in cancer therapy lead to an ever-increasing cancer cure rate. However, both RT and many chemotherapies are associated with side effects due to the injury of normal (non-cancer) tissues that are sometimes severe and almost always reduce the quality of life of cancer patients and survivors. From a clinical perspective, the dose of ionizing radiation that can be delivered is limited by the radiation tolerance of normal tissue [2]. RT of tumors in the chest may lead to the exposure of the whole or part of the heart to radiation. As a result, studies have been reported on the incidence of heart disease in ~10% or more of patients treated with RT for lung cancer, esophageal cancer, Hodgkin’s lymphoma, and breast cancer [3,4,5,6]. An improved understanding of normal tissue injury, such as in the heart, is required to develop pharmaceutical interventions that increase the safety of cancer therapy [7].

Investigations in pre-clinical research models can contribute to our understanding of mechanisms by which ionizing radiation injures the heart and may point to new targets for intervention [8,9]. In RT of thoracic cancers, the heart may only be partially exposed to radiation [10,11] and a radiation exposure to certain parts of the heart may be associated with a different cardiac outcome [12,13]. While previous X-ray technologies limited small animal models to whole heart or whole chest irradiation, recent developments have enabled image-guided partial heart X-ray exposures [14,15,16]. These prior studies have shown that a partial heart irradiation in mouse models leads to changes in cardiac function and that certain parts of the mouse heart may be specifically radiation-sensitive.

Mast cells are normally present in the heart in low numbers. However, in rat models of whole heart irradiation, the cardiac mast cell numbers increase and correlate with a radiation-induced collagen deposition in the myocardium [17,18,19,20]. Therefore, increased cardiac mast cell numbers may be an indication of radiation-induced adverse remodeling in the heart.

Toll-like receptors (TLRs) are pattern recognition receptors that recognize microbial components and endogenous ligands such as the extracellular matrix components and molecules released due to cellular injury. In complex with other transmembrane support molecules, including MD-2 [myeloid differentiation-2] and MyD88 [myeloid differentiation primary response 88], TLRs activate intracellular signaling pathways and induce the transcription of genes required for inflammatory responses. However, studies in various disease models, including in the heart, implicate that the inappropriate activation of the TLR signaling pathways results in deleterious inflammation and injury [21]. Of all TLRs, TLR4 has the highest expression in both the murine and human heart [22]. Studies have shown a role for TLR4 in myocardial inflammation due to myocardial infarction [23], myocarditis [24], and in heart failure [25]. TLR4 also regulates the production of cytokines in mast cells [26]. TLR4 inhibitors or knock-out approaches have been shown to reduce cardiac injury in many animal models, including cardiac dysfunction due to trauma hemorrhage [27]. However, while TLR4 can promote the production of collagen by cells in the heart [28], the inhibition of TLR4 does not always reduce myocardial fibrosis in cardiac disease models [29]. Moreover, despite the extensive knowledge of the role of TLR4 in cardiac disease, its role in radiation-induced heart disease is largely unknown.

In this study, we examined the effects of whole heart and partial heart irradiation on the cardiac collagen deposition, mast cell numbers, and left ventricular expression of TLR4 in male and female wild-type mice. In this model, adverse tissue remodeling occurred in both the irradiated and unirradiated portions of the heart.

## 2. Materials and Methods

### 2.1. Animal Housing

All procedures were approved by the Institutional Animal Care and Use Committee of the University of Arkansas for Medical Sciences (UAMS) under protocol numbers 3955 and TR202200000004. Thirty-two male and 32 female C57BL/6J mice were obtained from the Jackson Laboratories (Bar Harbor, ME, USA) at the age of 8 weeks and housed at the UAMS Division of Laboratory Animal Medicine, 4 mice per cage on a ventilated cage rack system, on a 12:12 light-to-dark cycle with free access to standard rodent chow and water.

### 2.2. Animal Irradiation

After one week of acclimatization, mice were exposed to whole or partial heart irradiation. For this purpose, mice were anesthetized with a 1–2% isoflurane inhalation and placed supine on a mouse platform in the Small Animal Radiation Research Platform (SARRP, XStrahl, Surrey, UK). Anesthesia was maintained during irradiation via a nosecone. A cone beam computed tomography (CBCT) scan was obtained of each animal (60 kV, 0.8 mA) and reconstructed using 720 projections. The SARRP Muriplan software was used to plan an X-ray beam (220 kV, 13 mA, 0.5 mm copper) locally to the heart while minimizing the exposure of the lungs and spinal cord. For whole heart irradiation, one beam (8 × 8 mm beam size) was delivered, with the gantry at −50 degrees and the couch at 75 degrees. A single dose of 8 or 16 Gy was applied. For the irradiation of the bottom 40% of the heart, one beam (5 × 5 beam size) at the same gantry and couch settings was executed, and a single dose of 16 Gy was administered. Sham-treated animals (0 Gy) were anesthetized and placed on the SARRP platform but not exposed to radiation. Example treatment plans and dose volume histograms are shown in Figure S1.

### 2.3. Plasma Collection

At 5 days and 2 weeks after irradiation, a peripheral blood sample was collected from each mouse. The facial vein was punctured, and 200–250 μL blood was collected into an EDTA-coated tube, immediately centrifuged at 1000× *g* for 15 min, and the plasma was snap-frozen and stored for metabolomics analysis.

### 2.4. Tissue Collection

At 6 months after irradiation, animals were anesthetized with 3% isoflurane inhalation, administered 30–40 U/kg of heparin, and the tissues were collected and immediately processed. The hearts were cut longitudinally, and one half of the heart was fixed in methanol Carnoy’s solution (60% methanol, 30% chloroform, 10% acetic acid). The remainder of the heart was dissected into the atria, left, and right ventricles and snap-frozen before their storage at −80 °C. In collecting the specimens of the left ventricle, care was taken to obtain specimens of the irradiated as well as the unirradiated parts of the heart.

### 2.5. Histology

All analyses were performed blinded to the experimental groups. For the determination of the collagen deposition, the sections of the heart were deparaffinized, rehydrated, and incubated in Sirius Red supplemented with Fast Green. The sections were scanned with a ScanScope CS2 slide scanner and analyzed with ImageScope 12 software (Aperio, Leica Biosystems, Buffalo Grove, IL, USA) to determine the percentage of the tissue area which was positive for collagens. The top half and the bottom half of the hearts were examined separately.

Mast cells are normally present in the heart in low numbers. However, in rat models of local heart irradiation, the cardiac mast cell numbers increase and correlate with the radiation-induced collagen deposition in the myocardium [17,18,19,20]. Therefore, increased cardiac mast cell numbers may be an indication of radiation-induced adverse remodeling in the heart. To visualize mast cells, the deparaffinized and rehydrated sections of the heart were incubated in 0.5% Toluidine Blue in 0.5 N HCl for 72 h, followed by 0.7 N HCl for 10 min. The mast cells were counted using an Axioskop transmitted light microscope (Zeiss International, Oberkochen, Germany) and divided by the total tissue area.

### 2.6. Western-Blotting

Samples of the frozen left ventricle of both irradiated and unirradiated parts of the heart were homogenized with a Potter-Elvehjem mechanical compact stirrer (BDC2002, Caframo LabSolutions, Georgian Bluffs, ON, Canada) in a 1% Triton-X100 radioimmunoprecipitation assay buffer containing protease inhibitors (1:100) and phosphatase inhibitors (1:100; Sigma-Aldrich, St. Louis, MO, USA). The protein concentration was determined with a bicinchoninic acid protein assay (Bio-Rad, Hercules, CA, USA), and 25 µg of protein was added to a 2× Laemmli buffer containing β-mercaptoethanol (5%). Gel electrophoresis was performed, and the proteins were transferred to a polyvinylidene difluoride membrane. The membranes were incubated in mouse anti-TLR4/MD2 complex (MTS510, Thermo Fisher Scientific, Waltham, MA, USA) or mouse anti-glyceraldehyde 3-phosphate dehydrogenase (GAPDH) (6C5, Santa Cruz Biotechnology, Santa Cruz, CA, USA) in TBS containing 0.1% Tween-20 and 5% non-fat dry milk at 4 °C overnight. After incubating with horseradish peroxidase-conjugated goat anti-mouse IgG (Jackson Immunoresearch, West Grove, PA, USA), the membranes were covered in enhanced chemiluminescence Plus Western Blotting Detection Reagent (GE Healthcare Life Sciences, Chicago, IL, USA) and placed on a CL-Xposure Film (Thermo Scientific, Waltham, MA, USA). The films were developed and imaged with an AlphaImager^®^ gel documentation system (ProteinSimple, San Jose, CA, USA). Densitometry was performed with ImageJ software. TLR4 was normalized to the loading control GAPDH and calculated relative to the expression of TLR4 in the sham-irradiated controls.

### 2.7. Metabolomics

The plasma samples collected at 5 days and 2 weeks were subjected to targeted metabolomics using liquid chromatography-mass spectrometry on a QTRAP 5500 (Sciex, Framingham, MA, USA). A detailed description of the procedures and statistical analysis is provided in Table S2.

### 2.8. Statistical Analysis

The metabolomics data were transformed using a log transformation to stabilize the variance, and a statistical analysis was performed using the unpaired *t*-test to determine the differential expression between the groups. Multiple testing correction was applied using the Benjamini–Hochberg procedure. Metabolites were identified that were 2-fold or more up-regulated or down-regulated at a *p* < 0.05 in the plasma of irradiated animals compared to the time- and sex-matched sham-irradiated controls. All other data of the male and female animals were combined and evaluated with a one-way analysis of variance (ANOVA) followed by the Tukey–Kramer test for multiple comparisons, using the software package NCSS 8 (NCSS, Kaysville, UT, USA). The criterion for significance was *p* < 0.05. With this analysis approach, the study had 80% power to detect an effect size (detectable contrast/standard deviation) of 1.5. The data are reported as individual values and the mean ± standard deviation.

## 3. Results

### 3.1. Histology

**Figure 1:**
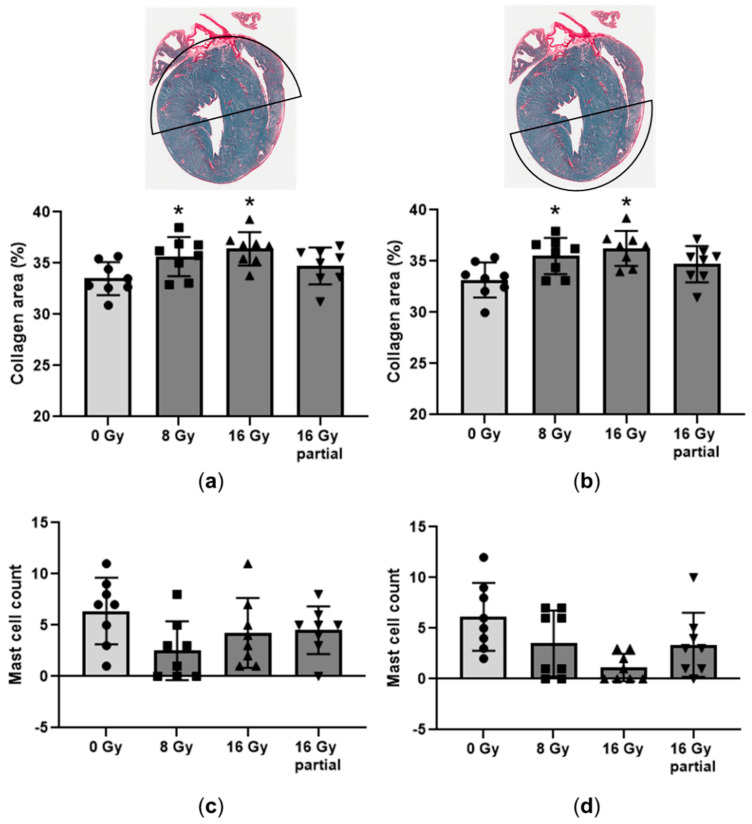
Cardiac histology 6 months after irradiation. Cardiac tissue area occupied by collagens was assessed (**a**) in the top half of the heart and (**b**) in the bottom half of the heart. Cardiac mast cell counts were determined (**c**) in the top of the heart and (**d**) in the bottom of the heart. Male and female data are combined, *n* = 8 animals per group. * *p* < 0.05 compared to 0 Gy.

The deposition of cardiac collagen did not seem to differ between males and females (Figure S2). Therefore, in Figure 1, the data from both sexes were combined. There was an increased collagen deposition 6 months after whole heart irradiation. There was no significant difference in the collagen deposition between the partially irradiated hearts and the sham-controls. Interestingly, this increase in the collagen deposition was seen in both the irradiated and unirradiated parts of the heart.

After high doses of radiation to the heart, the cardiac mast cell numbers correlate with the radiation-induced collagen deposition [17,18,19,20]. Therefore, we determined the numbers of mast cells in the heart. Radiation did not upregulate the mast cell count in either part of the heart (Figure 1).

### 3.2. Left Ventricular TLR4 Expression

**Figure 2:**
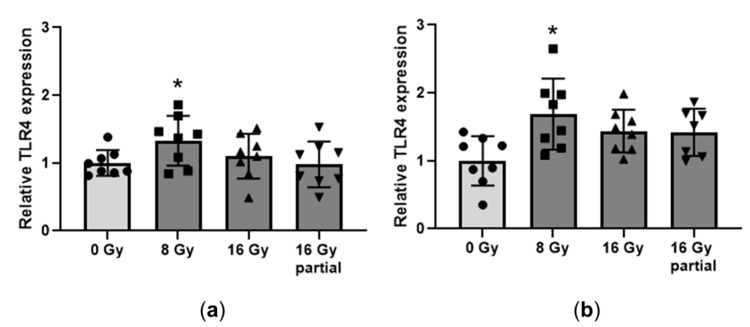
Left ventricular protein levels of TLR4. Western blotting was performed on specimens of the left ventricle in the top part of the heart (**a**) and the bottom part of the heart (**b**). Male and female data are combined, *n* = 7–8 animals per group. * *p* < 0.05 compared to 0 Gy.

Specimens of the left ventricular tissue were obtained from the top and bottom half of each heart and used to assess the protein levels of TLR4. There seemed to be local differences in the expression of TLR4, with a significant radiation-induced increase only in the bottom part of the left ventricle, after a dose of 8 Gy (Figure 2, Figure S3).

### 3.3. Plasma Metabolomics

A metabolomics analysis of the plasma samples was performed at 5 days and 2 weeks after the irradiation and comparisons were made with time- and sex-matched sham-irradiated controls. Raw data from the analysis are provided in Table S1. The analysis identified a total of 51 metabolites that were two-fold or more up-regulated or down-regulated at a *p* < 0.05 in the plasma of animals in one or more of the irradiation groups (Table S2). These metabolites may provide an indication of early post-irradiation metabolic changes in the hearts. Most of the significantly altered metabolites were identified at the day 5 time point. At this time point, all male irradiation groups showed a larger number of dysregulated metabolites than the females. A total of 12 metabolites were dysregulated in two irradiation groups or time points: epinephrine, glucosamine-6-phosphate, guanosine monophosphate, guanosine diphosphate, hippurate, homocysteic acid, 3-hydroxyisovaleric acid, 5-hydroxyl-indole-3-acetic acid, IDP, methylmalonate, succinate, and succinyl-CoA. One metabolite, L-ornithine, was upregulated in three irradiation groups (Table S2).

## 4. Discussion

Prior studies have investigated the effects of a partial heart irradiation on cardiac function in mouse models [14,15,16]. Ghita et al. showed more severe changes in cardiac function and structure as measured with echocardiography in C57BL/6J mice that were exposed to radiation to the base of the heart compared to the middle portion or the apex [14]. Lee et al. exposed a larger volume of the mouse heart to X-rays, in 25 fractions of 2 Gy, to mimic the tangential field RT in conventional fractions as in the clinic. This radiation protocol caused small changes in the cardiac function as measured with a pressure-volume loop method, mainly in ApoE^−/−^ mice [15]. Dreyfuss et al. exposed the apex of the heart of C57BL/6 mice to high single doses of radiation and documented radiation-induced fibrosis in the irradiated portion of the heart [16]. All three studies were performed in female mice.

The current study investigated the histological changes and left ventricular expression of TLR4 in male and female C57BL/6J mice that were exposed to a single-dose whole heart irradiation, or the irradiation of only the bottom 40% of the heart. We found a small increase in the deposition of cardiac collagen in the whole heart-irradiated mice, but not after a 16 Gy partial heart exposure. This suggests that in mouse models of partial heart irradiation, high doses of radiation are needed to induce fibrosis [16]. Although 6 months is a common post-irradiation time point at which radiation-induced cardiac fibrosis is examined in rodent models, we cannot exclude that a difference in the collagen deposition would have become apparent at a longer follow-up time. Moreover, in the current study, we did not have the opportunity to measure cardiac function, which is a major limitation, since cardiac radiation fibrosis may not be directly related to changes in the cardiac function.

Within the first 30 days after high doses of radiation to the heart, the cardiac mast cell numbers decrease, while after about 30 days, the mast cell numbers tend to increase above the sham-irradiated controls [17,20]. Cardiac mast cells can play both pro- and anti-fibrotic roles in the heart [30]. While the exact role of the mast cells in radiation-induced heart disease is not yet known, prior studies in mast cell-deficient rats suggest that mast cells may play a predominantly protective role in radiation-induced myocardial fibrosis [31]. In this study, the cardiac mast cell numbers were assessed at 6 months after whole or partial heart irradiation. Therefore, the study design was not suited to determine whether radiation induced a decrease in the cardiac mast cell numbers within the first month, as seen in prior studies. Moreover, at 6 months, no radiation-induced increases in the cardiac mast cell counts were seen, suggesting that mild radiation fibrosis in the heart is not associated with a mast cell infiltration.

The metabolomics analysis of the plasma samples collected at 5 days and 2 weeks after irradiation provided indications of early post-irradiation metabolic changes in the heart. Some of the metabolites were found to be up- or down-regulated in multiple irradiation groups or time points. Of those metabolites, epinephrine is known to enhance the production of nitric oxide in the endothelium [32]. L-ornithine, which was up-regulated in the plasma of three radiation groups, may be an indication of the increased activity of the enzyme arginase, which cleaves L-arginine to form L-ornithine and release urea. The reduced availability of L-arginine may cause endothelial dysfunction [33]. However, in the current study, the plasma levels of L-arginine in irradiated female mice showed a modest 1.4–1.6-fold increase (*p* < 0.05) compared to sham-irradiated female controls at 2 weeks (Table S1). Homocysteic acid is one of the main agonists of the N-methyl-D-aspartate receptor and may be involved in cardiac oxidative stress [34]. 5-hydroxyl-indole-3-acetic acid is a metabolite of serotonin and a potential biomarker of radiation injury [35]. Additional studies are required to determine whether plasma serotonin is an early marker of a radiation injury in the heart. Altered levels of guanosine monophosphate and guanosine diphosphate may be an indication of an altered energy requirement. Lastly, changes in succinate and succinyl-CoA may be an indication of an altered use of substrate in cardiac mitochondria, in line with our prior studies showing a decrease in succinate-driven state 2 respiration in mitochondria isolated from the irradiated rat heart [36].

Whether there are differences in the development of radiation-induced heart disease in males and females is largely unknown. While the number of mice per experimental group in this study was low, we saw no differences in the radiation-induced collagen deposition between male and female mice. On the other hand, at the day 5 time point, we identified a larger number of dysregulated metabolites in the plasma of irradiated males compared to females. Interestingly, TLR4 may be one of the inflammatory mediators that is differentially regulated in male and female subjects [37]. TLR4 recognizes bacterial and viral products, but also damaged self-tissue and plays a central role in inflammation in the heart [38]. Mast cells are among the immune cells that are regulated by TLR4 [39,40]. Further studies are required to understand radiation-induced inflammation in the heart, determine whether sex differences occur, and test whether TLR4 may be a target for intervention in radiation-induced heart disease.

## 5. Conclusions

This study shows early changes in plasma metabolites in mouse models of a whole heart and partial heart irradiation. The results obtained at 6 months after the irradiation suggest that adverse tissue remodeling may not only occur in the irradiated portion of the heart. While there was no indication of a difference between male and female mice in the cardiac collagen deposition or mast cell numbers, studies with larger numbers of animals per experimental group are required to study the sex effects in more detail.

## Data Availability

The data presented in this study are available as supplementary material.

## Supplementary Materials

The following supporting information can be downloaded at: https://www.mdpi.com/article/10.3390/cancers15020406/s1, Figure S1: Example treatment planning for mouse whole and partial heart irradiation; Figure S2: Cardiac tissue area occupied by collagens in male and female mice, in the top half of the heart (top panel) and bottom half of the heart (bottom panel); Figure S3: Left ventricular expression of TLR4 in male and female mice, in the top half of the heart (top panel) and in the bottom half of the heart (bottom panel); Table S1: Analysis of plasma metabolomics at 5 days and 2 weeks after whole heart or partial heart irradiation in male and female mice compared to time- and sex-matched sham-irradiated controls; Table S2: Metabolomics methodology and summary of significantly up- and down-regulated metabolites in plasma (*p* < 0.02 and fold change >2) at 5 days and 2 weeks after whole heart or partial heart irradiation.
