# Supplementary material for: Effects of Whole and Partial Heart Irradiation on Collagen, Mast Cells, and Toll-like Receptor 4 in the Mouse Heart
Source: Cancers (Basel). 2023 Jan 7;15(2):406. doi: 10.3390/cancers15020406 (PMC9856613; doi:10.3390/cancers15020406)
Supplement: Supplementary file 1 [file cancers-15-00406-s001.zip › Table S2.pdf]

Full methods for targeted metabolomics of plasma samples:

Mass spectrometry solvents and reagents: All LC-MS grade solvents including acetonitrile and water were purchased from Fisher Optima grade, Fisher Scientific. High purity formic acid (99%) was purchased from Thermo-Scientific. Debrisoquine and 4-nitrobenzoic acid were purchased from Sigma-Aldrich.

Sample Preparation and data acquisition details: The targeted metabolomics method was developed in-house to quantitate 270 endogenous molecules using QTRAP® 5500 LC-MS/MS System (Sciex, MA, USA). For this purpose, 25 µL of each plasma sample was dissolved in 100 µL of extraction buffer (methanol/water 50/50) containing 200 ng/mL of debrisoquine (DBQ) as internal standard for positive mode and 200 ng/mL of 4-nitrobenzoic acid as internal standard for negative mode. The samples were vortexed for 30 seconds and incubated on ice for 20 min. To the above mixture, 100 µL of chilled acetonitrile was added. The samples were incubated at -20 °C for 20 min. Samples were centrifuged at 13,000 rpm for 20 min at 4 °C. The supernatant was transferred to MS vial for LC-MS analysis. 5 µL of the prepared sample was injected onto a Kinetex 2.6 µm 100 Å 100 × 2.1 mm (Phenomenex, CA, USA) using SIL-30 AC auto sampler (Shimazdu, Kytoto, Japan) connected with a high flow LC-30AD solvent delivery unit (Shimazdu, Kytoto, Ja-pan) and CBM-20A communication bus module (Shimazdu, Kytoto, Japan) online with QTRAP 5500 (Sciex, MA, USA) operating in positive and negative ion mode. A binary sol-vent comprising of water with 0.1% formic acid (solvent A) and acetonitrile with 0.1% formic acid (solvent B) was used. The extracted metabolites were resolved at 0.2 mL/min flow rate. The LC gradient conditions were as follows: Initial – 100% A, 0% B for 2.1 minutes; 14 minutes – 5% A, 95% B till 15 minutes; 15.1 minutes – 100% A, 0% B till 22.5 minutes. The auto sampler and oven were kept at 15 °C and 30 °C, respectively. Source and gas setting for the method were as follow: curtain gas = 35, CAD gas = medium, ion spray voltage = 2500 V in positive mode and −4500 V in negative mode, temperature = 400 °C, nebulizing gas = 60 and heater gas = 70. The reproducibility and high quality of the LC-MS data were ensured using several measures. The column was conditioned using the pooled quality control (QC) samples initially, and pooled QC samples were also injected after every 10 sample injections to monitor shifts in signal intensities and retention time as measures of reproducibility and data quality of the LC-MS data. Pooled QC samples were routinely applied to assess and correct for any analytical variance. Blank solvent runs between sets of samples (after every 10 samples before and after pooled QC samples) were used to monitor sample-to-sample carry-over effects. To monitor instrumental variance, we ran NIST plasma sample (every 20 samples) prepared in the same manner as actual samples.

Data processing and statistical analysis: The abundance measurement for metabolites were expressed as intensity units that were initially normalized to internal standards and processed using MultiQuant 3.0.3 (Sciex). The data were pre-processed using a signal/noise ratio >20:1 and retention time tolerance of 5 seconds, after manually checking of metabolites peak by experts to find the reliable features. Thereafter, we used 20% of the coefficient of variation as our filter criteria to remove any possible noises before data normalization. Analytical drifts (if any) were corrected by quality control based robust LOESS signal correction (QC-RLSC). The data were then transformed using a log transformation to stabilize the variance. Statistical analysis was performed using the un-paired t-test, with a constraint of p-value <0.05, to determine differential expression be-tween the various groups. Multiple testing correction was applied using the Benjamini-Hochberg procedure. Metabolites were identified that were 2-fold or more up-regulated or down-regulated at a p<0.05 in the plasma of irradiated animals compared to time- and sex-matched sham-irradiated controls. Results are summarized in **Table S2** below.

Table S2: Significantly up- and down-regulated metabolites in plasma of male and female irradiated mice compared to time- and sex-matched sham-irradiated controls, at 5 days and 2 weeks after whole heart or partial heart irradiation.

	5 days						2 weeks					
	**Female 16 Gy**	**Male 16 Gy**	**Female 16 Gy PH**	**Male 16 Gy PH**	**Female 8 Gy**	**Male 8 Gy**	**Female 16 Gy**	**Male 16 Gy**	**Female 16 Gy PH**	**Male 16 Gy PH**	**Female 8 Gy**	**Male 8 Gy**
Acetoacetate	↓											
N-Acetylglycine	↓											
N-Acetyltyrosine						↑						
Aminosugar Phosphate				↑								
Amino Sugars		↓										
Biliverdin				↓								
Butylcarnitine								↑				
Citraconic Acid				↓								
L-Dihydroorotic Acid				↑								
2,3-Diphosphate-D-Glyceric Acid				↑								
Epinephrine		↓		↓								
Erythronic Acid				↑								
GABA					↓							
Geranyl-Pyrophosphate								↓				
Glucosamine 6-Phosphate				↑								↑
Guanidineacetate	↓											
Guanosine Diphosphate											↓	↓
Guanosine Monophosphate							↑		↑			
Guanosine Triphosphate				↓								
Hexanoylglycine						↓						
Hippurate		↓					↓					
Homocysteic acid				↑		↑						
3-Hydroxycinnaminic Acid												↓
3-Hydroxyisovaleric Acid		↓		↓								
5-Hydroxyl-indole-3-acetic acid		↑		↑								
2-Hydroxy-3-methylbutyric acid		↓										
IDP									↑		↑	

3-Indolebutyric acid	↓											
3-Indoxyl Sulfate		↓										
Isovaleric acid	↓											
Itaconic acid				↓								
Methionine Sulfoxide				↑								
Methylmalonate		↓		↓								
5-Methyluridine		↓										
3-Nitrotyrosine				↑								
Octadiecadienoylcarnitine						↓						
Oh-Phenylpyruvate		↓										
L-Ornithine				↑		↑					↑	
2-Oxobutanoate	↓											
Phenylacetylglycine										↑		
6-Phosphogluconate				↑								
Proline				↑								
Pyridoxal-5-Posphate											↑	
Pyridoxamine				↑								
D-Ribose-5-Phosphate				↑								
Spermidine				↓								
Succinate		↓		↓								
Succinyl-coA					↑						↓	
Tetradecadienoylcarnitine						↓						
Deoxythymidine Monophosphate					↑							
UDP-Glucose										↓		

PH: partial heart irradiation

↑ and ↓: significantly up- and down-regulated compared to time- and sex-matched sham-irradiated control ($p<0.05$, fold change>2)
