# Supplementary figures and images for: Effects of Whole and Partial Heart Irradiation on Collagen, Mast Cells, and Toll-like Receptor 4 in the Mouse Heart
Source: Cancers (Basel). 2023 Jan 7;15(2):406. doi: 10.3390/cancers15020406 (PMC9856613; doi:10.3390/cancers15020406)

Figure S1

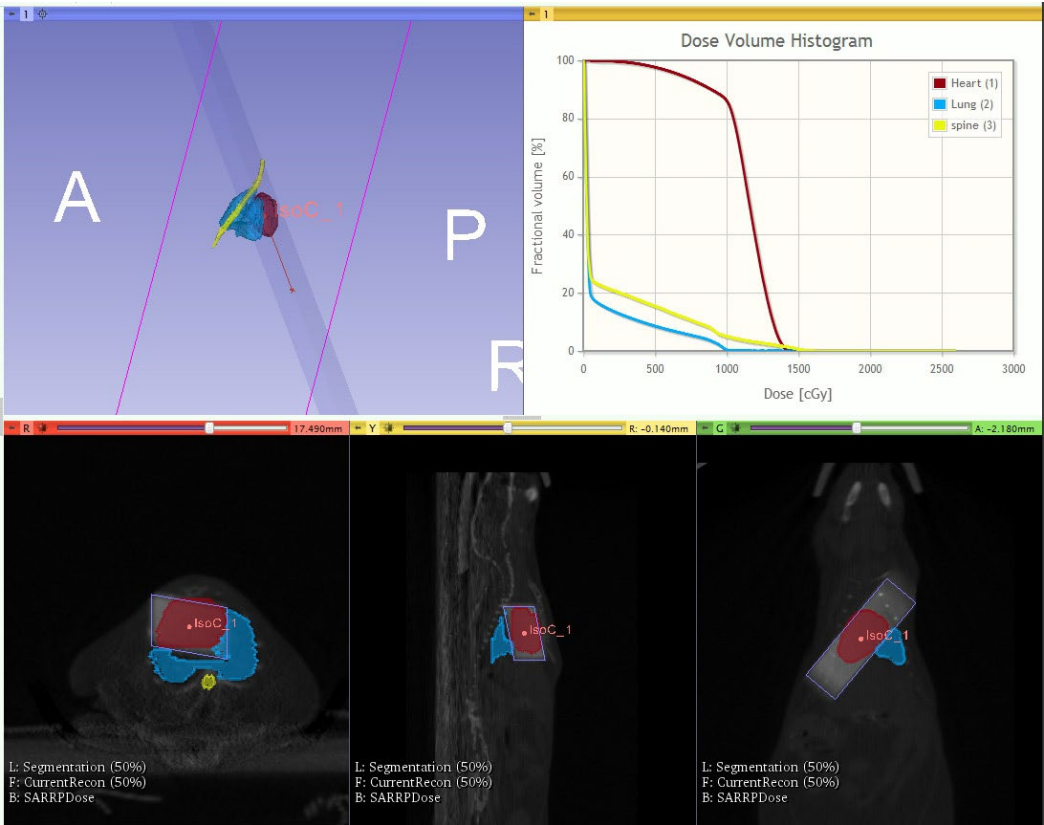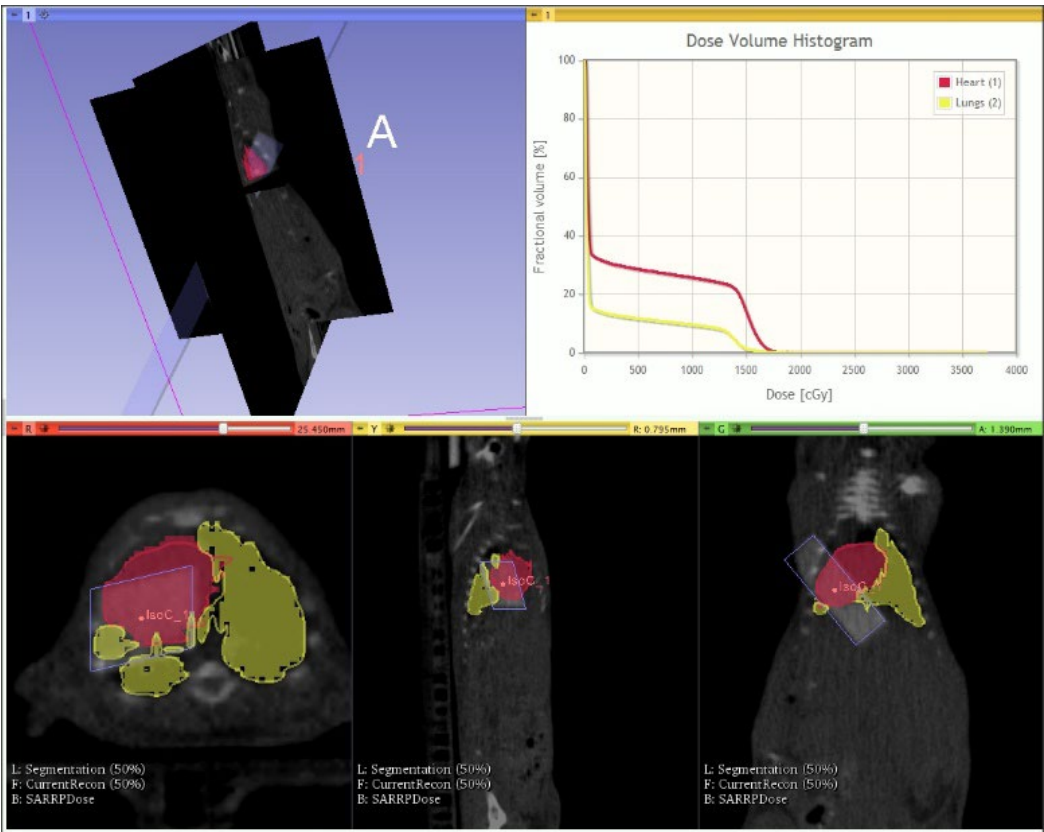

Supplement: Supplementary file 1 [file cancers-15-00406-s001.zip › Figure S1.pdf]

Figure S2

Top part of the heart

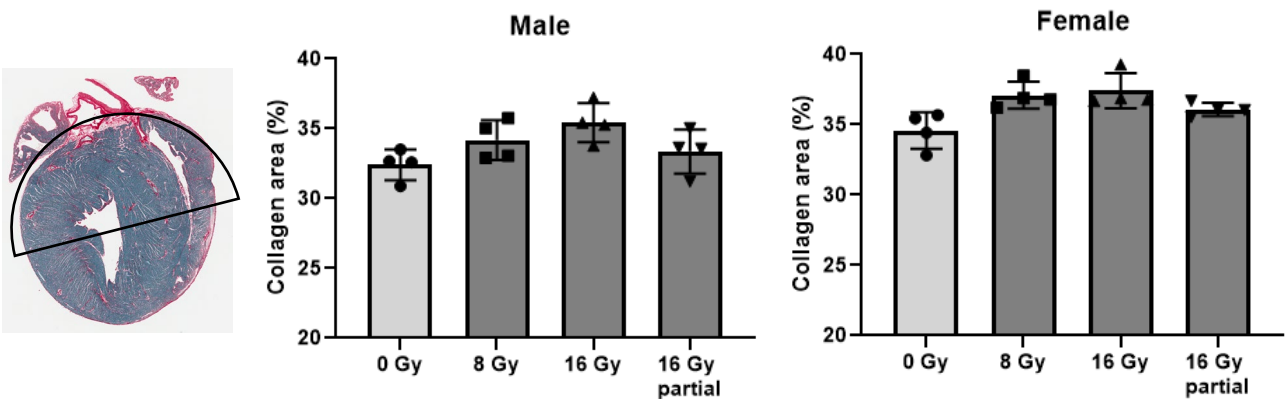

Bottom part of the heart

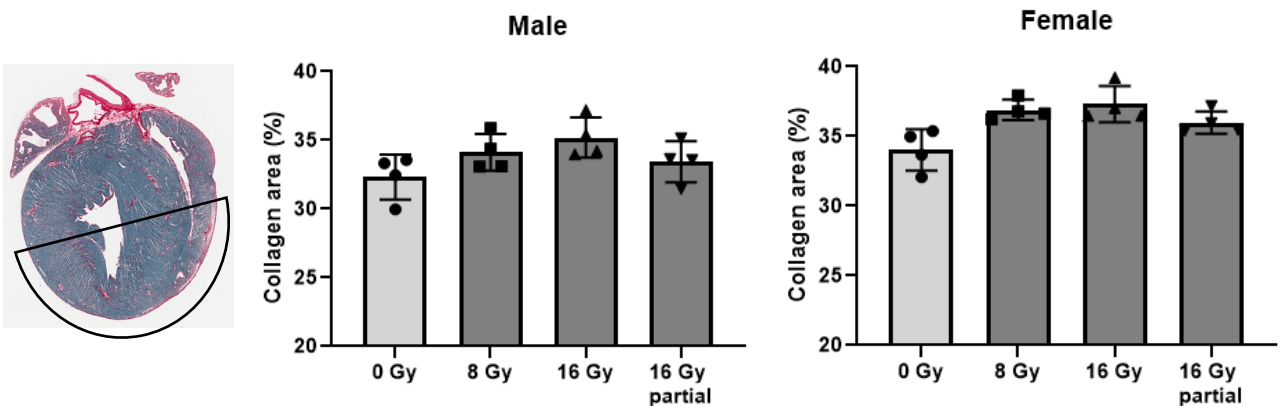

Supplement: Supplementary file 1 [file cancers-15-00406-s001.zip › Figure S2.pdf]

**Figure S3**

**Top part of the heart**

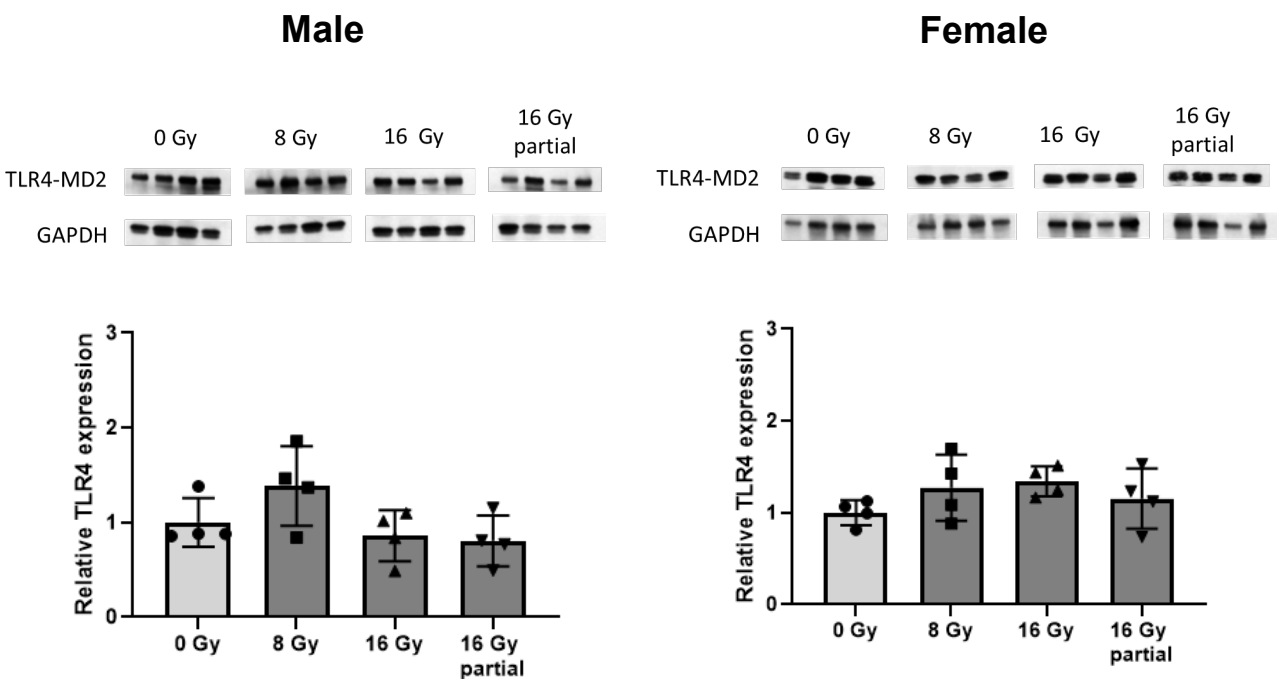

**Bottom part of the heart**

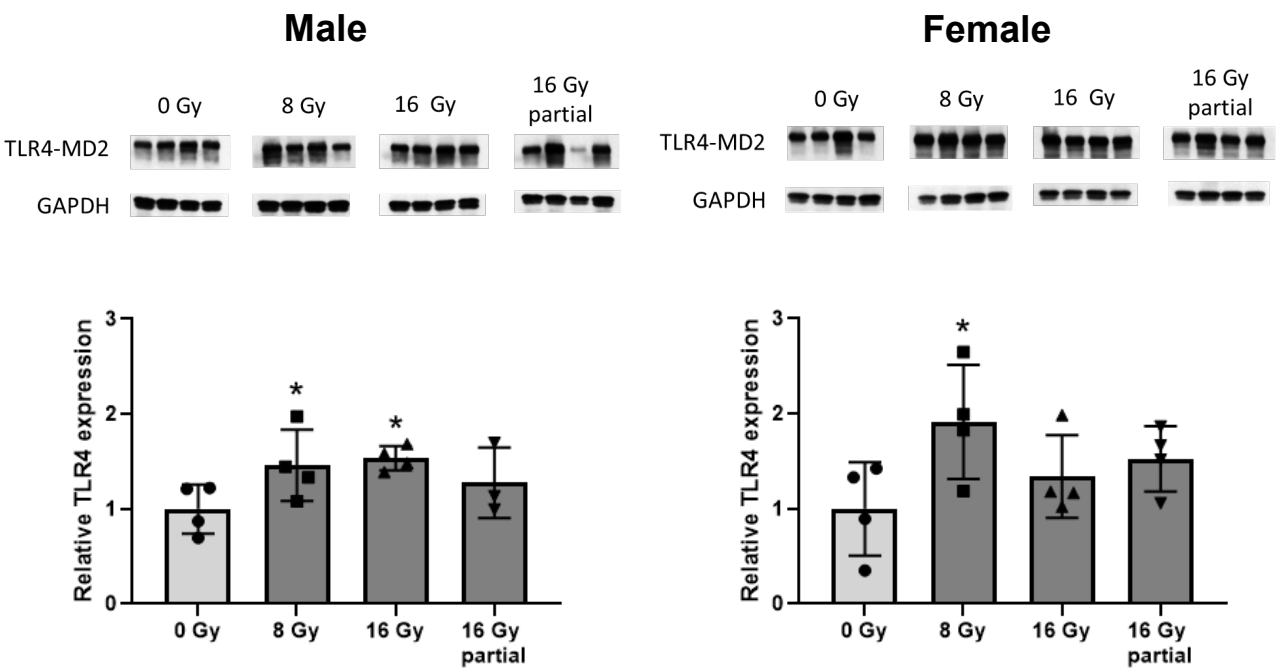

Supplement: Supplementary file 1 [file cancers-15-00406-s001.zip › Figure S3.pdf]
